# Compassion fatigue impacted by traumatic birth and psychological resilience among midwives in mainland China: an explanatory sequential mixed-methods

**DOI:** 10.1186/s12912-026-05120-x

**Published:** 2026-07-27

**Authors:** Di Tian, Rui Xia, Hui Li, Yao Yang, Yuxia Lin, Jinqiong He, Weilin Lin, Jin Li, Yuan Yang, Ying Zhou, Yulan Ren, Xi Su, Jiamei Li

**Affiliations:** 1 Guangzhou Medical University, No.1 Xinzao Road, Xinzao Town, Panyu District, Guangzhou, Guangdong 511436 China; 2 The Second Affiliated Hospital of Xuzhou Medical University, Xuzhou, Jiangsu 221006 China; 3 Shenzhen Baoan Women’s and Children’s Hospital, No.56 Yulu Road, Baoan District, Shenzhen, Guangdong 518133 China; 4 The Eighth Affiliated Hospital of Southern Medical University (The First People’s Hospital of Shunde), Foshan, Guangdong 528308 China; 5 State Key Laboratory of Ophthalmology, Zhongshan Ophthalmic Center, Sun Yat-sen University, Guangzhou, Guangdong 510180 China

**Keywords:** Compassion fatigue, Traumatic birth, Psychological resilience, Midwives, Mixed methods

## Abstract

**Background:**

Compassion fatigue (CF) among midwives threatens both their well-being and the quality of maternal care. This study examined how traumatic birth exposure, psychological resilience, and work-family factors influence CF among midwives in mainland China.

**Methods:**

An explanatory sequential mixed-methods design was employed. Phase 1 involved a cross-sectional survey of 227 midwives from eight public hospitals in Guangzhou, using the Professional Quality of Life Scale (ProQOL) and Connor-Davidson Resilience Scale (CD-RISC). Multivariable ordinal logistic regression identified predictors of CF. Phase 2 included 15 in-depth interviews to contextualize quantitative findings, with data analyzed thematically.

**Results:**

Nearly all participants (98.7%) reported some degree of CF, with over half experiencing moderate or higher levels. Higher psychological resilience significantly reduced CF severity (OR = 0.888, 95% CI: 0.851–0.926, *p* < 0.001), while sole childcare responsibility (OR = 4.442, 95% CI: 1.439–13.722, *p* = 0.010), longer professional experience, extended shifts, and frequent traumatic birth exposure (≥ 3 events/month) increased CF risk. Moderate traumatic exposure (1–2 events/month) was protective compared to no exposure. Qualitative data revealed mechanisms such as emotional burden, recovery needs, and organizational support.

**Conclusions:**

CF is prevalent among Chinese midwives and influenced by traumatic birth exposure, resilience, and work-family demands. Interventions should integrate organizational reforms (e.g., shorter shifts, debriefing) with resilience training and peer support to mitigate CF and sustain care quality.

**Supplementary Information:**

The online version contains supplementary material available at https://doi.org/10.1186/s12912-026-05120-x.

## Background

*The 2021 State of the World’s Midwifery report* (SoWMy 2021) highlighted significant progress in the profession while also drawing attention to emerging challenges for the midwifery workforce [1]. Although midwives are universally recognized as critical to maternal and newborn health, many now face increasing pressures, including exhaustion, burnout, and work-related trauma [2, 3]. A growing body of research confirms that midwives worldwide experience high levels of compassion fatigue (CF) [4, 5]. Given midwives’ vital role in safeguarding mothers and infants, preserving their psychological well-being is an urgent global priority.

CF among midwives has attracted attention due to its many negative consequences. At the individual level, CF is linked to reduced job satisfaction, depression, anxiety, moral distress, and a weakened professional identity [5]. At the health system level, CF among providers can undermine the quality of maternal and newborn care and strain relationships with patients [6, 7]. At the societal level, CF contributes to workforce shortages and increased healthcare costs, impeding national efforts to improve maternal health [2, 8]. Therefore, addressing midwives’ CF is critical for attracting and retaining skilled professionals and for ensuring the quality of maternal care nationwide.

Crowe (2006) defined CF as the emotional, moral, and physical distress that can occur from caring for and witnessing others’ suffering [9]. Stamm (2010) conceptualized professional quality of life (ProQOL) in three components: compassion satisfaction (CS), burnout (BO), and secondary traumatic stress (STS), which together reflect the positive and negative aspects of caregiving [10]. CS refers to the sense of pleasure, achievement, and professional value derived from helping others, and is regarded as the positive dimension of professional quality of life [10]. In contrast, BO and STS represent the negative dimensions of professional quality of life. BO is generally characterized by physical and emotional exhaustion, reduced professional efficacy, and psychological detachment from work, and is closely related to prolonged occupational stress and adverse work environments [11]. Among midwives, heavy workload, long or irregular shifts, staffing pressure, workplace conflict, and insufficient organizational support may increase vulnerability to BO [12]. Previous studies have shown that burnout among midwives is associated with reduced job satisfaction, intention to leave, actual turnover, and compromised quality and continuity of maternal and newborn care [13]. STS arises from caring for individuals who have experienced traumatic or stressful events, leading to emotional distress in the caregiver [14]. Prior studies report that CS is inversely related to burnout and STS, while burnout and STS are positively associated [2, 15]. In the context of midwifery, exposure to traumatic birth events may increase STS and burnout, decrease compassion satisfaction, and thereby contribute to heightened CF [16]. Understanding midwives’ traumatic experiences may therefore be crucial to addressing CF in this population.

Exposure to traumatic birth events can increase healthcare providers’ risk of CF [17]. As frontline maternal care providers, midwives frequently encounter unpredictable challenges, including traumatic births [18]. For instance, one study reported that experiencing a traumatic birth was a key factor exacerbating CF among midwives [19]. Other research found that approximately 85–94% of midwives have experienced at least one traumatic birth event in their careers, and a greater frequency of such events was associated with higher CF [14]. In China, the recent implementation of the “three-child policy” (2021) has led to more obstetric complications (e.g., due to advanced maternal age), likely increasing the incidence of traumatic births [20]. These trends suggest growing challenges for Chinese midwives in managing CF.

Research suggests that individuals with stronger psychological resilience tend to recover more quickly from traumatic events than those with lower resilience [21]. Resilience refers to an individual’s capacity to adapt to adversity. Studies among nurses have found that resilience is inversely related to CF [6, 22]. However, while CF has been documented among midwives internationally, the combined influence of traumatic birth experiences and personal resilience on midwives’ CF has not been well studied. To fill this gap, the present study examines the psychological resilience of midwives in general hospitals in Guangzhou, China, and explores how resilience and traumatic birth events affect their levels of CF.

### Theoretical framework

Kumpfer’s Resilience Framework served as the theoretical foundation for this study [23]. This framework emphasizes the dynamic interaction between external environmental stressors, such as work conditions and family support, and internal resilience resources, including cognitive, emotional, and behavioral competencies. Stressful events are viewed as triggers that activate resilience processes. Through ongoing person-environment interactions, these processes lead to outcomes ranging from successful adaptation to maladaptive responses.

**Fig. 1:**
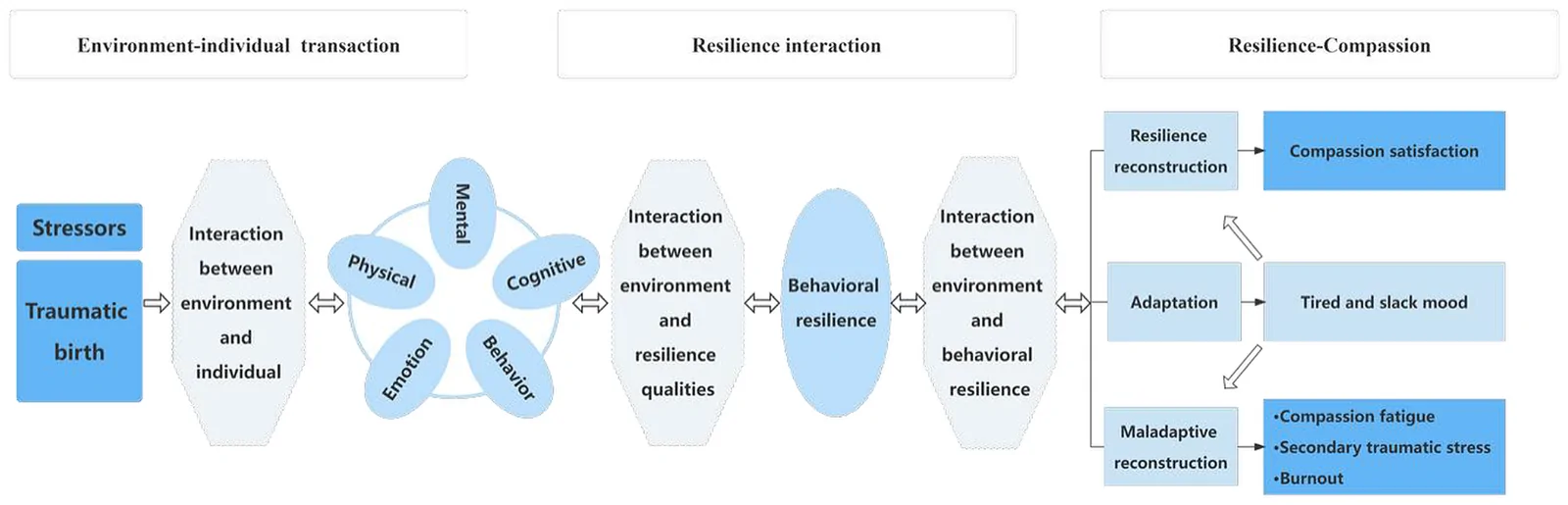
The resilience framework of this study

Grounded in this model, the current study conceptualized traumatic birth experiences and work-environment stressors as external stressors, and psychological resilience as an internal protective resource. These factors may influence midwives’ professional quality of life, specifically compassion satisfaction, burnout, and secondary traumatic stress, as illustrated in Fig. 1. Although traumatic birth experiences are closely related to STS, their impact on midwives may not be limited to trauma-specific symptoms. Repeated exposure to obstetric emergencies and adverse birth outcomes may also contribute to chronic occupational exhaustion, reduced professional efficacy, and diminished CS. Therefore, examining CF within the professional quality of life framework provides a broader perspective for understanding how traumatic birth experiences and work-environment stressors jointly affect midwives’ psychological well-being and quality of care.

The study aimed to address two key questions: (1) What are the prevalence and main determinants of CF among midwives? (2) How do traumatic birth experiences and psychological resilience interact to influence CF, and what underlying coping mechanisms explain these relationships?

### Methods

#### Study design

**Fig. 2:**
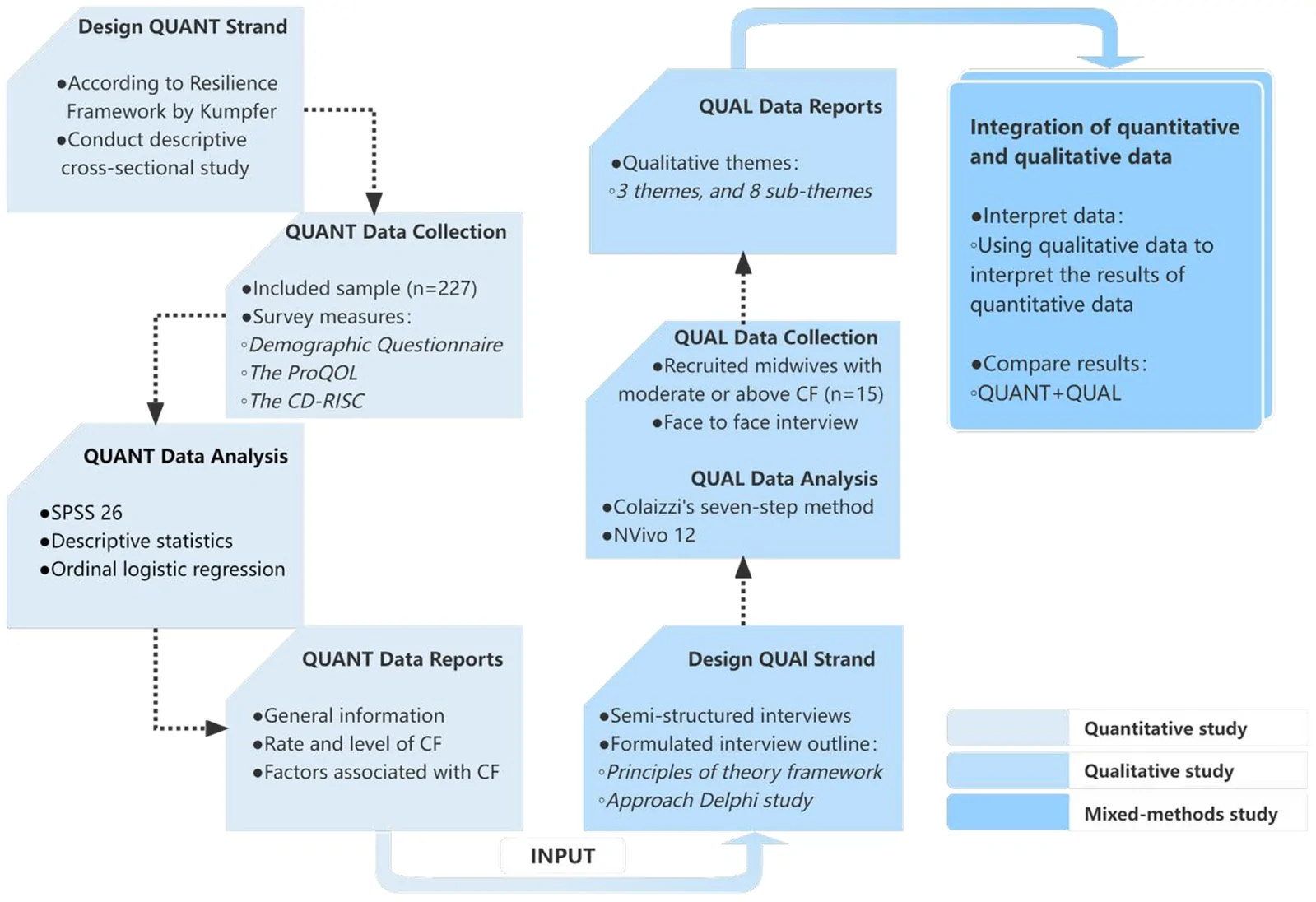
Diagram for the explanatory sequential design

This study employed an explanatory sequential mixed-methods design (QUANT → QUAL) to comprehensively investigate CF among midwives [24]. Reporting followed GRAMMS guidance [25]. The study was conducted in two sequential phases: (1) Phase 1 (quantitative study): A cross-sectional survey was first administered to a sample of midwives to assess the prevalence of CF and quantitatively examine its associations with traumatic birth exposure, psychological resilience, and other demographic and occupational factors. (2) Phase 2 (qualitative study): Subsequently, in-depth semi-structured interviews were conducted with a purposively selected subset of survey participants. This phase aimed to elaborate, contextualize, and provide mechanistic explanations for the key quantitative findings. The overall flow of the study is illustrated in Fig. 2. The two phases were connected through purposive sampling, refinement of the interview guide, and integrated interpretation. Preliminary quantitative findings guided the selection of interview participants and informed the development of qualitative probing questions, while qualitative findings were subsequently used to explain and contextualize the statistical associations.

### Participants and recruitment

Midwives were recruited via convenience sampling from eight public hospitals of comprehensive universities in Guangzhou, China. Eligibility criteria required participants to: (1) hold both a Chinese nurse license and a maternal and infant care license, (2) be currently on clinical duty, and (3) be willing to participate. Interns and those working full-time in administration, management, teaching, or research were excluded. The sample size for the quantitative phase was estimated based on the conventional requirement of at least 10 observations per predictor variable for multivariable regression [26]. With 20 candidate predictors, at least 200 valid observations were required. To allow for an anticipated 20% rate of nonresponse or invalid questionnaires, 240 participants were targeted. After questionnaire distribution and data screening, 227 valid responses were included in the final analysis, satisfying the minimum sample size requirement. For the qualitative phase, 15 participants were purposively selected from Phase 1 respondents who scored at least in the moderate range for CF, ensuring variation in key characteristics (e.g., hospital, years of experience). Recruitment continued until thematic saturation was achieved.

### Data collection instruments and procedures

Quantitative Data Collection (May – December 2023): Participants who met the eligibility criteria and provided consent completed a self-administered questionnaire packet, which required approximately 20 min. The packet included: (1) Socio-demographic Questionnaire: This questionnaire gathered information on midwives’ demographic and occupational characteristics, including experiences of traumatic birth, work environment, workload, salary, childbearing status, and sleep quality etc. (2) Professional Quality of Life Scale (ProQOL): The 30-item ProQOL scale was used to assess CS, BO, and STS, each subscale comprising 10 items rated on a 5-point Likert scale (1–5). Subscale scores were categorized as low (≤ 22), moderate (23–41), or high (≥ 42). Cronbach’s *α* values for the three subscales were 0.88, 0.75, and 0.81, respectively [10]. The Chinese ProQOL version was translated by Chen & Wang in 2012 [27] and has been validated in several studies. Its Cronbach’s *α* values for the three subscales were 0.82, 0.73, and 0.76, respectively. Because the ProQOL manual scores CS, BO, and STS as three separate subscales and does not provide a standardized overall CF severity score, CF severity in this study was further operationalized using a Chinese scoring approach that has been applied in previous nursing research [27]. In this approach, the risk criteria for the three ProQOL subscales were defined as low CS (CS < 37), high BO (BO > 27), and high STS (STS > 17). Participants were then categorized into CF severity levels according to the number of criteria met: no criterion indicating no CF, one criterion indicating mild CF, two criteria indicating moderate CF, and three criteria indicating severe CF. In this classification, CS was treated as an inverse indicator, with lower CS representing reduced positive professional quality of life. Although this classification is not part of the original ProQOL scoring manual, it has been applied in Chinese nursing research to capture the multidimensional nature of CF. (3) Connor–Davidson Resilience Scale (CD-RISC): Psychological resilience was measured using the 25-item CD-RISC, comprising three domains (tenacity, strength, and optimism) with items scored 0–4; higher total scores indicate greater resilience; the Cronbach’s *α* value was 0.89 [28]. The Chinese version of the CD-RISC was translated and verified by Xiaonan Yu in 2007; its Cronbach’s *α* value was 0.91 [29].

Qualitative Data Collection (January – March 2024): Face-to-face, semi-structured interviews were conducted with the 15 selected participants. The interview guide was developed through a sequential process. First, an initial guide was drafted based on Kumpfer’s Resilience Framework, focusing on traumatic birth experiences, psychological resilience, work-family demands, and work-environment factors. Second, after preliminary quantitative analysis, the guide was refined to strengthen the connection between the quantitative and qualitative phases. Key quantitative findings, including significant associations and unexpected patterns, informed additional probing questions about emotional responses, recovery, professional confidence, burnout, compassion satisfaction, and coping resources. Finally, the revised guide was reviewed through a Delphi consultation with five senior experts in maternity and psychological care to enhance its content validity [30]. Details of the expert authority coefficients and interview outline are provided in Supplementary Table S1 and S2. Interviews were conducted in a private setting by two researchers: one leading the interview and the other observing and documenting nonverbal cues. Sessions were audio-recorded with consent, supplemented by field notes, and typically lasted 20 min.

### Data analysis

Quantitative analysis: Data were analyzed using SPSS 26 (IBM, Armonk, NY, USA). Continuous variables were summarized as means with standard deviations, and categorical variables as frequencies with percentages. Respondents were classified into none, mild, moderate, or severe CF. Potential predictors included socio-demographic factors, psychological resilience, and traumatic birth experience. Given the ordinal outcome, ordinal logistic regression (with severe CF as the reference) was applied, and odds ratios (OR) with 95% confidence intervals (CI) were reported; *p* < 0.05 was considered significant.

Qualitative Analysis: Interviews were transcribed verbatim within 24 h and analyzed using Colaizzi’s seven-step phenomenological method [31] with NVivo 12. The analysis involved repeatedly reading the transcripts, extracting significant statements, formulating meanings, and organizing themes and subthemes. To enhance trustworthiness, two researchers independently coded the transcripts, and coding discrepancies were discussed within the research team until consensus was reached. Member checking was conducted by returning summarized meanings or preliminary interpretations to selected participants to confirm the accuracy of the findings. An audit trail, including interview notes, coding records, analytic memos, and team discussion summaries, was maintained throughout the analysis. Reflexive discussions were also conducted to minimize the influence of researchers’ preconceptions.

Quantitative regression identified statistical associations, while qualitative themes provided explanatory narratives for these relationships, clarifying underlying mechanisms and contextual factors.

## Results

### Phase 1 — Quantitative findings

A total of 240 questionnaires were distributed and 235 returned (response rate 97.9%); after excluding eight incomplete forms, 227 valid responses were analyzed (effective rate 94.6%). Participants were all female; mean age was 33.34 ± 8.71 years (range 23–56), mean professional experience was 10.82 ± 8.92 years (range 1–35), and 83% were married. Other sample characteristics are summarized in Table 1.

**Table 1:** Factors associated with compassion fatigue among midwives using an ordinal logistic regression model (*n* = 227)

Variable	Category	*n* (%)	β	SE	Wald χ2	*P* value	Odds ratio (OR)	95% CI
Number of night shifts per month	0	13(5.7%)	0.767	0.799	0.921	0.337	2.153	0.450-10.309
	1–2	15(6.6%)	2.362	1.053	5.029	0.025*	10.612	1.347–83.680
	3–4	17(7.5%)	− 0.102	1.069	0.009	0.924	0.903	0.111–7.338
	> 4	182(80.2%)	0^a^	.	.	.		
Child care giver	Others	94(41.4%)	1.491	0.575	6.721	0.010*	4.442	1.439–13.722
	Self	133(58.6%)	0^a^	.	.	.		
Satisfaction with salary	Extraordinary dissatisfaction	3(1.3%)	0.147	2.250	0.004	0.948	1.158	0.014–95.297
	Dissatisfaction	30(13.2%)	1.055	1.548	0.464	0.496	2.872	0.138–59.680
	Common	134(59.0%)	− 0.182	1.433	0.016	0.899	0.834	0.050-13.832
	Satisfaction	56(24.7%)	− 0.243	1.451	0.028	0.867	0.784	0.046–13.477
	Extraordinary satisfaction	4(1.8%)	0^a^	.	.	.		
Pressure of competition	Less	9(4.0%)	− 0.302	1.049	0.083	0.773	0.739	0.095–5.778
	Normal	147(64.8%)	0.839	0.465	3.263	0.071	2.314	0.931–5.755
	Excessive	73(31.2%)	0^a^	.	.	.		
Professional title	Primary	139(61.2%)	− 0.590	1.406	0.176	0.675	0.554	0.035–8.715
	Intermediate	80(35.3%)	0.799	0.582	1.884	0.170	2.223	0.710–6.959
	Senior	8(3.5%)	0^a^	.	.	.		
Expert outpatient service	Yes	53(23.3%)	0.770	0.519	2.200	0.138	2.160	0.780–5.977
	No	174(76.7%)	0^a^	.	.	.		
Monthly income (RMB)	< 5000	34(15.0%)	0.321	0.624	0.264	0.607	1.379	0.405–4.683
	5000–9999	114(50.2%)	0.637	0.471	1.833	0.176	1.891	0.752–4.759
	> 10,000	79(34.8%)	0^a^	.	.	.		
Sleep quality	Normal	49(21.6%)	0.534	0.622	0.739	0.390	1.706	0.505–5.772
	Occasional sleeplessness	129(56.8%)	0.467	0.495	0.891	0.345	1.595	0.605–4.208
	Frequently sleeplessness	49(21.6%)	0^a^	.	.	.		
Work on pre- and post-birth services	None	95(41.9%)	− 0.504	0.490	1.058	0.304	0.604	0.231–1.578
	< 20%	40(17.6%)	0.377	0.666	0.321	0.571	1.458	0.395–5.382
	20–40%	18(7.9%)	-1.041	0.732	2.025	0.155	0.353	0.084–1.481
	40–60%	21(9.3%)	− 0.033	0.551	0.004	0.952	0.968	0.328–2.849
	> 60%	53(23.3%)	0^a^	.	.	.		
Fertility status	Nulliparous	90(39.6%)	0.281	0.878	0.102	0.749	1.324	0.237–7.396
	Parous	137(60.4%)	0^a^	.	.	.		
Education level	Secondary	4(1.8%)	− 0.262	1.550	0.029	0.866	0.770	0.037–16.071
	Junior	18(7.9%)	0.206	0.715	0.083	0.773	1.229	0.303–4.983
	Graduate and above	205(90.3%)	0^a^	.	.	.		
Working environment	Satisfactory	127(55.9%)	-1.085	1.492	0.529	0.467	0.338	0.018–6.297
	Common	95(41.9%)	0.154	1.460	0.011	0.916	1.166	0.067–20.369
	Dissatisfactory	5(2.2%)	0^a^	.	.	.		
Working years			0.126	0.049	6.658	0.010*	1.134	1.030–1.249
Age			− 0.026	0.052	0.245	0.621	0.974	0.880–1.079
Take a vacation	Delayed	143(63.0%)	− 0.059	0.400	0.022	0.882	0.943	0.430–2.063
	On time	84(37.0%)	0^a^	.	.	.		
Psychological resilience			− 0.119	0.022	30.678	0.000*	0.888	0.851–0.926
Number of traumatic births experienced per month	> 4	77(33.9%)	− 0.403	1.152	0.122	0.726	0.668	0.070–6.392
	3–4	133(58.6%)	− 0.178	0.970	0.034	0.854	0.837	0.125–5.601
	1–2	9(4.0%)	− 0.924	0.413	4.999	0.025*	0.397	0.176–0.892
	0	8(3.5%)	0^a^	.	.	.		
Marital status	Single	82(36.1%)	− 0.477	0.776	0.378	0.539	0.621	0.136–2.838
	Married	145(63.9%)	0^a^	.	.	.		
Workload	Appropriate	127(55.9%)	− 0.545	0.431	1.602	0.206	0.580	0.249–1.349
	Excessive	100(44.1%)	0^a^	.	.	.		
Working hours per shift	Appropriate	169(74.4%)	-1.014	0.468	4.697	0.030*	0.363	0.145–0.908
	Excessive	58(25.6%)	0^a^	.	.	.		

(*p < 0.05; 0^a^ means reference)

#### Prevalence and scale scores

Nearly all respondents (224/227, 98.7%) reported some degree of CF. Mean ProQOL subscale scores were: CS 36.10 ± 5.26, BO 24.76 ± 4.23, and STS 26.17 ± 4.68 — all falling in the moderate range. Mean CD-RISC score was 62.39 ± 11.94, indicating moderate resilience. Full score distributions and severity categories appear in Table 2.

**Table 2:** Incidence rate of compassion fatigue and mean scores for other domains (*n* = 227)

Variable	Frequency (%) / Mean ± SD
Degrees of compassion fatigue	
None	3(1.3%)
Mild	97(42.7%)
Moderate	78(34.4%)
Serious	49(21.6%)
Compassion fatigue	
Compassion satisfaction	36.10±5.26
Burnout	24.76±4.23
Secondary traumatic stress	26.17±4.68
Psychological resilience	62.39±11.94
Tenacity	31.35±6.54
Strength	21.40±4.24
Optimism	9.64±2.36

#### Predictors of compassion fatigue

Ordinal logistic regression identified several independent predictors of greater CF severity (Table 1). Higher psychological resilience was strongly protective: each one-point increase in CD-RISC reduced the odds of being in a more severe CF category by 11% (OR = 0.888, 95% CI: 0.851–0.926, *p* < 0.001). Family caregiving burden was a major risk factor: midwives who were sole caregivers for their children had more than fourfold higher odds of more severe CF than those with other caregivers (OR = 4.442, 95% CI: 1.439–13.722, *p* = 0.010). Longer cumulative professional experience modestly increased odds (OR = 1.134 per year, 95% CI: 1.030–1.249, *p* = 0.010).

#### Workload and shift patterns were also important

Working shifts of ≤ 8 h was associated with substantially lower odds of severe CF compared with extended shifts (OR = 0.363, 95% CI: 0.145–0.908, *p* = 0.030), indicating a protective effect of shorter shifts. A nonlinear pattern emerged for night-shift frequency: respondents reporting 1–2 night shifts/month had higher odds of severe CF relative to those with > 4 night shifts/month (OR = 10.612, 95% CI: 1.347–83.680, *p* = 0.025); this counterintuitive finding lacked an obvious explanation in the qualitative data. Traumatic-birth exposure showed a dose-response pattern: exposure to 1–2 events/month traumatic birth was associated with lower odds of severe CF versus no exposure (OR = 0.397, 95% CI : 0.176–0.892, *p* = 0.025), whereas frequent exposure (≥ 3 events/month) markedly increased odds. Collectively, these quantitative results indicate that resilience buffers risk while childcare burden, longer tenure, extended hours, certain night-shift patterns, and high traumatic-birth exposure increase CF risk.

### Phase 2 — Qualitative findings

15 midwives from five hospitals completed semi-structured interviews (total interview time = 335 min). Participant characteristics are presented in Table 3.

**Table 3:** Demographic and clinical characteristics of the interviewed midwives (*n* = 15)

Number	Age group (years)	Working years	Professional title	Level of compassion fatigue	Married	Parous
M1	30–39	10	Intermediate	Moderate	Yes	Yes
M2	20–29	6	Primary	Moderate	Yes	Yes
M3	20–29	5	Primary	Serious	No	No
M4	20–29	5	Primary	Moderate	Yes	Yes
M5	20–29	5	Primary	Serious	No	No
M6	20–29	3	Primary	Serious	No	No
M7	20–29	5	Primary	Moderate	No	No
M8	20–29	3	Primary	Serious	No	No
M9	20–29	3	Primary	Serious	No	No
M10	20–29	6	Primary	Serious	No	Yes
M11	20–29	4	Primary	Moderate	No	No
M12	30–39	10	Intermediate	Moderate	Yes	No
M13	30–39	8	Primary	Moderate	Yes	Yes
M14	30–39	7	Primary	Moderate	Yes	Yes
M15	30–39	10	Intermediate	Moderate	Yes	Yes

**Fig. 3:**
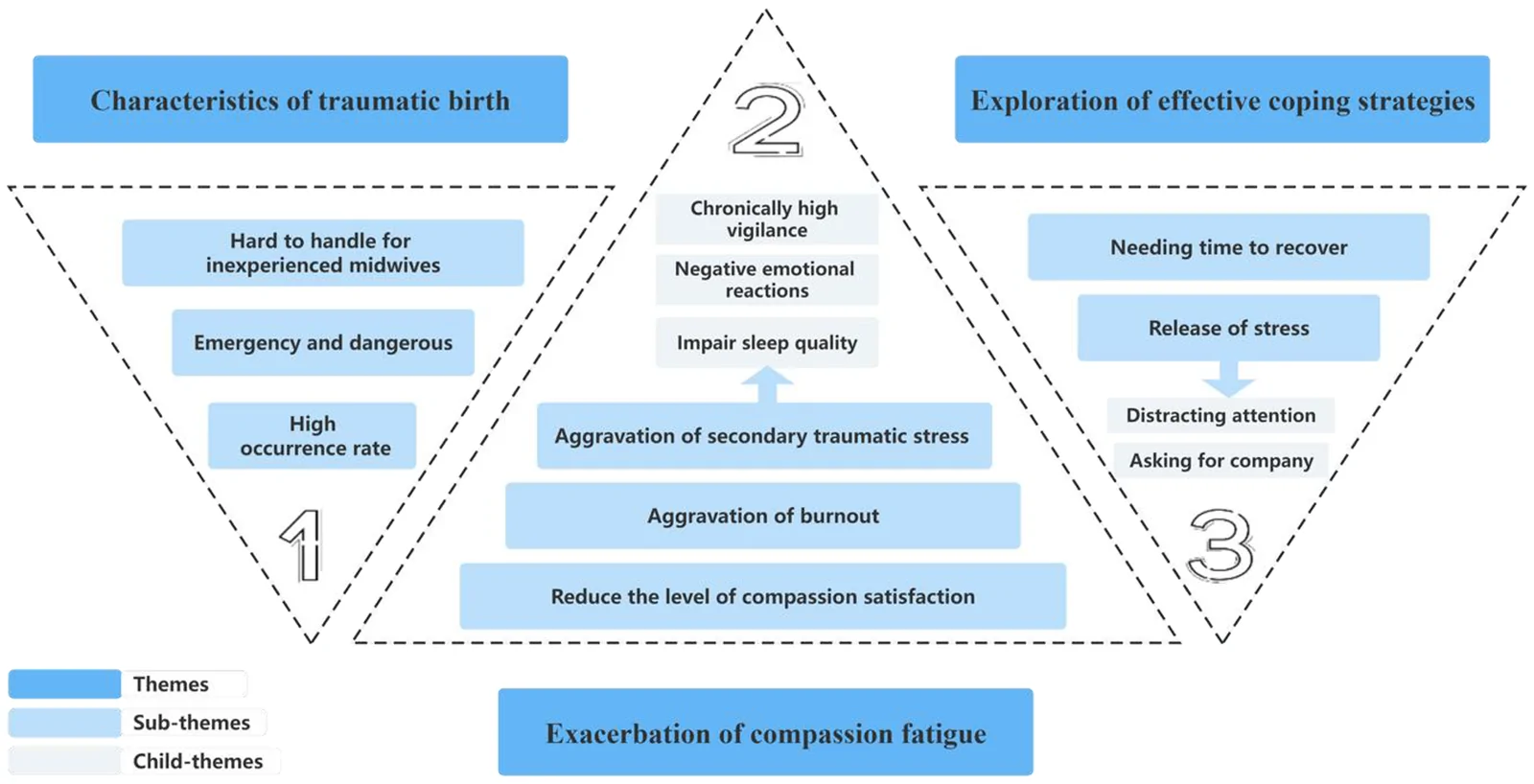
Thematic framework of qualitative findings

Thematic analysis yielded three primary themes and eight subthemes; representative quotations are provided in Supplementary Table S3, and the thematic framework is shown in Fig. 3.

### Theme 1: Stimulate characteristics of traumatic birth

#### High occurrence rate

Most interviewed midwives viewed traumatic birth as inevitable, attributing this to recent fertility policy changes and the rising number of advanced maternal age cases.

I think traumatic birth is common and unavoidable in our work, and you learn to adapt. (M5)

We’re in the clinical business, and it (traumatic birth) is certainly impossible to avoid. (M13)

#### Emergency and dangerous

The maternity ward is a complex battlefield; the situation of mother and baby changes rapidly. Traumatic birth occurs as an emergency, and the condition is dangerous.

The bleeding happened very quickly (postpartum), in just a minute or two. The woman had a pretty large pool of blood, and suddenly she went from a conscious state to a coma, and the change (postpartum situation) was very rapid” (very surprised expression). (M5)

#### Hard to handle for inexperienced midwives

Inexperienced midwives’ coping difficulties. Junior midwives lack the expertise to manage emergencies, while overexposure to traumatic births can lead to desensitization. Experiencing one or two traumatic birth events may build competence, but beyond that, it risks fostering apathy.

Now, of course, I think I have more experience. I am not so afraid, and I think I can deal with this thing (traumatic birth). I mean, your work years are growing, and the more you have seen, the more you will follow the process now when you encounter these kinds of things. So, there is no great worry (smile). (M4)

### Theme 2: Interacted dynamically-exacerbation of compassion fatigue

#### Reduce the level of compassion satisfaction

CS refers to the sense of satisfaction and efficacy generated in the process of helping people, which is an affirmation of self-worth [15]. The midwives were deeply frustrated by the traumatic birth. They had low satisfaction with the self-helping role and helping behavior, and a low level of CS.

The responsibility of midwives is to help puerperae give birth safely. But if your knowledge, skills, and work experience are not well equipped to help them, you will doubt your qualifications as a health care worker. (M8)

After such a traumatic birth, you will find that only midwifery skills are not enough. Your humanistic care ability, first-aid cooperation ability between doctors and nurses, and coordination ability between team members all have a lot of room for improvement. (M9)

#### Aggravation of burnout

Burnout refers to a constellation of symptoms arising from prolonged high pressure, including physical and emotional exhaustion, diminished professional efficacy, and withdrawal from work roles [32]. In our data, burnout often manifested after episodes of workplace blame, humiliation, or persistent stress, which undermined midwives’ motivation and led some to avoid clinical duties.

On one occasion, during the rescue of postpartum massive hemorrhage, there was hemolysis in a blood sample, and the doctor directly said that my blood drawing method was wrong, and did not consider the clotting situation of the puerperae, and then put the responsibility on me. I’m so afraid of offending the doctor that I don’t want to go to work (silent cry). (M5)

#### Aggravation of secondary traumatic stress

STS arises from witnessing or hearing about others’ trauma [14]. In our interviews, STS presented as immediate shock and acute negative emotions, followed by sleep disturbance and sustained hypervigilance. Negative emotional reactions. Traumatic births often provoked an initial stunned response that quickly became anxiety, sadness, or guilt.

1. Negative emotional reactions. Traumatic births often provoked an initial stunned response that quickly became anxiety, sadness, or guilt.

I was standing in the back watching the woman give birth to the placenta, and the blood came out like a faucet. I was stunned. I couldn’t think what to do (sigh). (M9)

A baby was born with severe asphyxiation. I felt very nervous, so I called for rescue immediately. (M3)

The baby died. I felt particularly sad, after all, it is a small life, and it is a particularly heavy blow to the mother and her family. I cried for several days after I came back because of this incident” (eyes were red and voice choked). (M9)

2. Impair sleep quality. Disturbing images recurred at night and disrupted sleep.

The dead child and the mother’s desperate and frightened expression may cross my mind suddenly before I go to sleep, so that I can’t sleep. (M11)

3. Sustained hypervigilance. Midwives described persistent self-monitoring and fear of missed signs after traumatic events.

It makes you have an obsessive thought about yourself. If I do nothing, for example, I do not evaluate changes in vital signs of puerperae, the mother and her baby might die. (M5)

### Theme 3: Remodeled exploration of effective coping strategies

#### Needing time to recover

Interviews linked recovery time to resilience: after a traumatic birth, most midwives said adjustment required days to weeks, sometimes longer.

It may take one to two months, and it scares me to think about it from time to time (forced smile). (M1)

It may take a few days to forget something like this, and then you will remember it from time to time, but the mood swings will not be so great. (M6)

#### Release of stress

Common coping strategies included distraction (exercise, travel, baking), seeking company, and reflective practices (e.g., journaling).

I just bake, eat, or go shopping, and when the pressure is too much, we will travel to distract ourselves. (M10)

Just self-regulation, sometimes I will write a diary. I am wondering, do you have any ways to help me? (M13)

### Integrated findings

Triangulation of quantitative and qualitative data clarified pathways linking exposure, context, and outcomes. Interview narratives illuminated key quantitative associations, including resilience as a protective factor and childcare burden, tenure, shift length, and traumatic birth exposure as risk factors. Qualitative accounts suggested mechanisms by which resilience operates, such as faster emotional recovery, use of adaptive coping strategies and meaning making, and they described how organizational and family demands erode opportunities for recovery.

Qualitative data further explained how years of service and salary dissatisfaction were connected to midwives’ subjective experiences of CF. Longer professional experience was described as a double-edged factor: it increased familiarity with emergency procedures and confidence in managing traumatic births, but it also reflected cumulative exposure to high-risk situations, responsibility, and emotional labor. Salary dissatisfaction was not discussed only as a financial issue; rather, it reflected participants’ perceived mismatch between workload, professional risk, emotional investment, and institutional recognition. These narratives helped explain why tenure and salary dissatisfaction may be linked to CF through chronic occupational strain and burnout-related experiences.

The finding that 1–2 traumatic birth events per month was related to lower odds of severe CF was contextualized by participants’ reports that moderate exposure can promote skill acquisition and measured confidence; conversely, frequent exposure overwhelmed coping capacity, producing intrusive recollections, sleep disruption, and reduced compassion satisfaction. One unexpected quantitative result, the substantially higher odds of severe CF in the 1–2 night shifts/month subgroup compared with > 4 shifts/month, was not directly explained by the qualitative data and warrants further investigation.

## Discussion

This mixed-methods study examined the prevalence and extent of CF among midwives in mainland China, highlighting how exposure to traumatic births, psychological resilience, and work–family conditions interact to influence these outcomes. Nearly all midwives exhibited some degree of CF, with more than half experiencing moderate or higher levels. This pattern aligns with findings from other contexts, such as a UK study where 67% of midwives reported moderate or above work-related burnout [13], and is consistent with a systematic review across 11 countries that reported an overall moderate level of CF among healthcare providers [15]. In our interviews, most midwives reported low satisfaction with their supportive care role. Persistent CF can lead to psychological and physical harm, contribute to workforce attrition, and ultimately exacerbate midwife shortages and compromise maternal care quality [32].

Guided by Kumpfer’s Resilience Framework [23], which proposes that stressors activate resilience processes leading to varied adaptation outcomes through person-environment interactions, our findings indicate that CF among midwives stems from the interplay between internal resources (particularly psychological resilience) and external stressors, such as traumatic births, long working hours, night shifts, and family care responsibilities. Psychological resilience served as a key protective factor, while traumatic birth exposure, extended working hours, and years of practice were salient external stressors. These findings are consistent with prior studies linking traumatic birth, work environment, tenure, work patterns, and social context to midwives’ CF [16, 33, 34]. Both quantitative and qualitative data converged on similar factors: protective elements included manageable shift lengths (≤ 8 h) and family support, whereas frequent traumatic births and excessive night shifts increased vulnerability.

Our findings suggest that CF among midwives may arise through two overlapping pathways. On the one hand, BO was mainly reflected in chronic occupational strain related to workload, shift patterns, cumulative years of service, perceived imbalance between effort and recognition, and limited recovery time. These conditions may gradually erode professional efficacy, emotional energy, and willingness to remain in clinical practice [5]. On the other hand, STS was more closely linked to trauma-specific reactions after witnessing severe obstetric emergencies or adverse maternal and neonatal outcomes, including intrusive memories, sleep disturbance, guilt, and sustained hypervigilance. Repeated traumatic birth exposure may therefore trigger STS-related symptoms, while a demanding and insufficiently supportive work environment may intensify BO and reduce compassion satisfaction [34]. These interacting processes highlight the need for interventions that combine trauma-informed post-event support with organizational strategies addressing workload, scheduling, peer support, and protected recovery time.

A key contribution of this study is identifying witnessing traumatic birth as a trigger for CF with a nonlinear relationship to monthly exposure. Midwives reporting 1–2 events/month had lower odds of severe CF than those with no exposure, whereas ≥ 3 events/month was linked to higher fatigue. Limited exposure may heighten vigilance and prompt preventive or restorative actions [35], and moderate exposure may promote experiential learning and confidence [36]. Qualitative interviews supported these mechanisms: occasional traumatic events were described as stressful but formative, while frequent exposure produced intrusive memories, sleep disturbance, and emotional numbing. This pattern does not imply that greater experience is protective; prior work links traumatic birth exposure to increased post-traumatic stress disorder risk [37], and potential retraumatization [38]. Repeated exposure may overwhelm psychological resilience, elevating CF risk. Thus, both zero and high (≥ 3 events/month) traumatic birth exposures emerged as significant risk factors. Interviews showed that despite the inevitability of traumatic births, midwives felt satisfaction and pride in managing emergencies and safeguarding mothers and infants, which helped mitigate regret. Overall, findings suggest a threshold effect: moderate exposure with recovery opportunities may support learning, while high cumulative exposure increases secondary traumatic stress and burnout, highlighting the importance of managing exposure and ensuring recovery time.

The study also underscores psychological resilience as a vital protective factor. Quantitatively, higher resilience was associated with lower CF severity. Qualitative findings further suggested that resilience may operate through both cognitive and behavioral processes. Cognitively, resilient midwives appeared more able to reappraise traumatic birth experiences as opportunities to strengthen clinical judgment, emergency preparedness, and professional competence, which may reduce persistent self-blame and support meaning-making. Behaviorally, they were more likely to seek peer or family support, discuss difficult cases with colleagues, engage in reflective practices such as journaling, and use leisure activities to release stress. These coping processes may facilitate emotional regulation, shorten recovery time, and help maintain compassion satisfaction after traumatic events. These findings are consistent with existing evidence that resilience can buffer the impact of occupational stressors on CF [39, 40]. However, resilience should not be viewed solely as an individual trait. Peer support, timely debriefing, supportive leadership, and protected recovery time may provide the conditions needed to sustain resilience [22]. Therefore, interventions should combine individual-level resilience training with organizational strategies that promote peer support and psychologically safe work environments [33, 34].

Beyond individual factors, external conditions significantly influenced midwives’ vulnerability to CF. Quantitatively, high workload and family care burdens, such as being the sole childcare provider or working extended shifts, increased the risk of severe fatigue, whereas shifts of 8 h or fewer were protective. Interviews further showed that long hours, night shifts, and childcare demands fueled exhaustion, while supportive environments, including family encouragement, peer support, and mentorship, promoted recovery and compassion satisfaction. These observations corroborate earlier studies on the importance of social and organizational support in fostering resilience among health professionals [7, 18], affirm that resilience is embedded in relational and organizational contexts. Therefore, systemic efforts involving government, health institutions, and hospitals are essential to implement supportive policies, ensure welfare protections, and cultivate workplace cultures that mitigate risk and promote resilience.

An unexpected finding was the higher odds ratio for CF among midwives working 1–2 night shifts/month compared to those with more than four. This may reflect a non-linear or contextual effect, as infrequent and irregular shifts could disrupt sleep and recovery more than predictable schedules, particularly for those with caregiving responsibilities or lower resilience. Evidence shows that night work disrupts circadian rhythms and sleep [41], while unstable schedules and short recovery periods are linked to poorer rest and psychological distress [42]. Caregiving roles may exacerbate these effects, whereas resilience can buffer them [43]. Future research should explore shift regularity, recovery time, and interactions with caregiving and resilience, with interventions prioritizing schedule predictability and protected recovery [44].

### Limitations and implications for practice

This study has several limitations. First, its cross-sectional design prevents causal inferences, and the sample from public hospitals in Guangzhou may limit generalizability to other regions or private settings. Second, although CF was operationalized within the ProQOL framework to capture the multidimensional negative professional quality of life involving BO, STS, and reduced CS, the use of a combined CF indicator may have limited our ability to isolate trauma-specific psychological processes related specifically to STS. Future studies should examine STS and BO separately, in addition to combined CF, to clarify their distinct and overlapping mechanisms. Third, the qualitative phase was voluntary and may therefore be subject to self-selection bias. Midwives who agreed to participate in interviews may have had particularly intense experiences, stronger motivation to express emotional distress, or, conversely, greater readiness to discuss traumatic events. Therefore, the qualitative findings may not fully represent the perspectives of all eligible midwives. Fourth, the prevalence and severity of CF may have been underestimated because midwives experiencing extreme BO or STS may have lacked the emotional capacity to complete the questionnaire or participate in interviews, and some may have already left clinical practice. This potential healthy-worker or survivor bias should be considered when interpreting the findings. Finally, excluding midwives in administrative or educational roles reduces representativeness, and the unexpected finding regarding night-shift frequency requires further investigation.

Despite these limitations, the findings highlight important practical implications. To reduce CF among midwives, interventions should address both trauma-specific and work-environment-related pathways. At the organizational level, hospitals should consider implementing predictable and manageable shift schedules, limiting excessively long shifts, ensuring protected recovery time after traumatic birth events, and providing structured post-event debriefing and psychological support. Workplaces should also strengthen peer support, mentorship, and psychologically safe communication, particularly after severe obstetric emergencies or adverse maternal and neonatal outcomes. At the individual level, integrating resilience training, stress-management strategies, and reflective practices into continuing professional development may help midwives improve emotional regulation, reduce isolation, and sustain compassion satisfaction. These combined strategies are essential for protecting midwives’ psychological well-being, supporting workforce retention, and maintaining the quality and continuity of maternal and newborn care.

## Conclusion

This explanatory sequential mixed-methods study found that midwives in mainland China commonly experience moderate CF, with resilience, traumatic birth exposure, childcare burden, and workload as key determinants. Qualitative interviews showed that repeated high-stress events and limited recovery erode compassion satisfaction, while resilience buffers these effects. In the context of the three-child policy, rising service demand may increase exposure and staffing pressures. To protect midwives’ well-being and care quality, we recommend resilience training, routine post-event psychological support, and system-level measures such as staffing and scheduling reforms that ensure recovery time. Aligning fertility policy goals with workforce investment is essential to safeguard caregivers and perinatal care.

## Supplementary Information

Below is the link to the electronic supplementary material.

Supplementary Material 1

## Data Availability

The datasets generated and analysed during the current study are not publicly available due to privacy and confidentiality reasons, but are available from the corresponding author on reasonable request.

## Abbreviations

CF: Compassion Fatigue
CS: Compassion Satisfaction
BO: Burnout
STS: Secondary Traumatic Stress
ProQOL: Professional Quality of Life Scale
CD-RISC: Connor–Davidson Resilience Scale
QUANT → QUAL: Quantitative followed by Qualitative (explanatory sequential design)
