# Supplementary material for: Compassion fatigue impacted by traumatic birth and psychological resilience among midwives in mainland China: an explanatory sequential mixed-methods
Source: BMC Nurs. 2026 Jul 27;25:999. doi: 10.1186/s12912-026-05120-x (PMC13685234; doi:10.1186/s12912-026-05120-x)
Supplement: Supplementary file 1 [file 12912_2026_5120_MOESM1_ESM.docx]

**Supplementary material**

**Table S1** Experts characteristics and results of the analysis of expert consultation

| Expert number | Professional field | Professional title | Education background | Authority coefficient of experts | | | **S-CVI** |
| --- | --- | --- | --- | --- | --- | --- | --- |
|  |  |  |  | **Ca** | **Cs** | **Cr^a^** |  |
| S1 | Midwifery | Senior | Master's degree | 1.05 | 1.00 | 1.03 | 1 |
| S2 | Midwifery | Senior | Master's degree | 1.10 | 1.00 | 1.05 |  |
| S3 | Critical care obstetrics | Associate senior | Master's degree | 1.10 | 1.00 | 1.05 |  |
| S4 | Obstetrics and gynecology、Psychology | Associate senior | Doctorate | 1.10 | 1.00 | 1.05 |  |
| S5 | Midwifery | Associate senior | Master's degree | 1.10 | 1.00 | 1.05 |  |

**^a^** Expert consultation results are reliable when the Cr ≥0.7.

**Table S2 The final semi-structured interview outline - in English language**

**Interview outline**

(1) What do you think about the characteristics of traumatic delivery

(2) Could you talk in detail about your inner feelings when experiencing a traumatic delivery event?

(3) How did it affect you physically and psychologically after experiencing a traumatic delivery event?

(4) How long does it take you to recover emotionally and mentally after a traumatic birth event? What approach was taken? Which works?

(5) Do you feel satisfied with your helping role and helping behavior? Please explain your reasons.

(6) Are you willing to continue working as a midwife after experiencing traumatic delivery? Please give your reasons.

(7) What would you like to add about midwifery and traumatic delivery events?

**Table S3** Representative quotes of the participants.

| **Themes and subthemes** | **Representative** | **Frequency** |
| --- | --- | --- |
| **Theme 1: Characteristics of traumatic delivery** | |  |
| **Subtheme 1: High occurrence rate** | Quote 1: Of course, a lot of things he will have a certain incidence of here, such as postpartum bleeding or, umbilical cord prolapse or, he will have a certain incidence of incidence. I would think that these events are impossible to avoid. (M9)  Quote 2: It (traumatic childbirth) could happen anytime, anywhere.When it comes to traumatic childbirth, it is inevitable, and you learn to adapt. (M5)  Quote 3: We're in the clinical business, and it (traumatic birth) is certainly impossible to avoid. (M13) | 21 |
| **Subtheme 2: Emergency and dangerous** | Quote 4: The bleeding happened very quickly (postpartum), in just a minute or two. The woman had a pretty large pool of blood, and suddenly she went from a conscious state to a coma, and the change (postpartum situation) was very rapid *“very surprised expression”*. (M5)  Quote 5: At that point, this (bleeding) was serious. There is the blood like water. (M6)  Quote 6: I was feeling very nervous and very scared. The real moment, when I watch his placenta come out and his blood comes out of the vagina like a tap, I think I'm stupid, you know. I watched the mother from a can communicate with your normal state of suddenly he went into shock, he was a little into a coma of that feeling! He can't open his eyes, he can't talk. (M9) | 22 |
| **Subtheme 3: Hard to handle for inexperienced midwives** | Quote 7: Now, of course, I think I have more experience. I am not so afraid and I think I can deal with this thing (traumatic birth). I mean, your work years are growing, and the more you have seen, you will follow the process now when you encounter this kind of things. So, there is no great worry(M4)  Quote 8: At the beginning, you won't be very worried, and you don't know what you should do. You just started, you are just a small employee. You may not even know what to do with this matter, or the leader will help you and teach you how to do it. After years of work, such a thing happened again, then you probably know how to go (deal with) repair this thing. (M7)  Quote 9: Then maybe at the beginning of the work, I will feel that I did not handle it well or some reasons or other reasons did not handle it well. Now after your analysis, you will know what to do better next time. I will sum up some lessons from it, let oneself do better next time. (M3)  Quote 10: If you will participate in the rescue process than before, you will be more and more confident, that is, I am more and more stable, I may from the beginning, is a nervous, fear, and then do not know what to do, the head is blank. Up to now, I think I can completely cope with this to cooperate or even to participate in the rescue, cooperate with the doctor, I think so far, I am very confident, I can do this, well. (M9) | 26 |
| **Theme 2: Exacerbating compassion fatigue** | |  |
| **Subtheme 1: Reducing the level of compassion satisfaction** | Quote 11:It's easy to be more irritable. I think because of this is in this state for a long time, if you are in this pressure for a long time, people's personality will change (the above reason). (M1)  Quote 12: The responsibility of midwives is to help puerperae birth safely. But if your knowledge, skills and work experience are not well equipped to help them, you will doubt your qualifications as a health care worker.I am disappointed, is just a big blow to my career. (M8)  Quote 13: After such a traumatic birth, you will find that only midwifery skills are not enough. Your humanistic care ability, first-aid cooperation ability between doctors and nurses, and coordination ability between team members all have a lot of room for improvement. (M9) | **30** |
| **Subtheme 2: Aggravated burnout** | Quote 14: Emotional it is easier to get out of control, more prone to insomnia, lack of sleep and so on, and then lack of sleep and lead to fatigue ah, and then work mental subsidies, concentration is not easy oh, service attitude and so on! It will have an impact. (M1)  Quote 15: On one occasion, during the rescue of postpartum massive hemorrhage, there was hemolysis in a blood sample, and the doctor directly said that my blood drawing method was wrong, and did not consider the clotting situation of the puerperae, and then put the responsibility on me. I'm so afraid of offending the doctor that I don't want to go to work “*silent cry”*. (M5)  Quote 16: More nervous, and then a little bit avoidant. I don't want to contact the patient again, and then I don't want to follow him up again. (M12) | **7** |
| **Subtheme 3: Aggravated secondary traumatic stress** |  |  |
| Negative emotional reactions | Quote 17: A baby was born with severe asphyxiation. I felt very nervous, then I called for rescue immediately. (M3)  Quote 18: I was standing in the back watching the woman give birth to the placenta, and the blood came out like a faucet. I was stunned. I couldn't think what to do “*sigh”*. (M9)  Quote 19: The baby died. I felt particularly sad, after all, it is a small life, and it is a particularly heavy blow to the mother and her family. I cried for several days after I came back because of this incident *“eyes were red and voice choked”.* (M9)  Quote 20: Midwives have more psychological pressure, and introverted people who refuse to talk will be more affected by traumatic childbirth events. (M11)  Quote 21:At that time, from the nurse to the director, all around me (accountability), and then I can't say what is the problem, I, I was like anyone off work, and very headache, and then sleepy, and then at that time is that what a face of confusion, this is a uh. I feel quite wronged. (M5) | 111 |
| Impaired sleep quality | Quote 22: The dead child and the mother’s desperate and frightened expression may cross my mind suddenly before I go to sleep, so that I can’t sleep. (M11)  Quote 23: Is also afraid ah, and then sometimes dream to sleep at night will imagine themselves lying on the operating bed, and then a very nervous very anxious state of mind er, and then I think a greater influence on me is to see more premature and pregnant women. Then I thought I had to make a little money before I thought about getting married and having children. (M6)  Quote 24: No matter how late I am, I may want to play 3,4 hours and so on this kind of situation, often get yourself to go to bed very late. Because you know that after the night shift for a long time, you tell me to sleep, I can sleep, but I can be awake no matter how sleepy I feel, and if I don't play this once a day, I feel uncomfortable. (M5) | 35 |
| Chronically high vigilance | Quote 25: It makes you have an obsessive thought about yourself. If I do nothing, for example, I did not evaluate changes in vital signs of puerperae, the mother and her baby might die. Feel very blank, won't produce what is that kind of perceptual understanding, is the feeling is dead, I think this really feel ah, here not well, and then there is to make up for the hole that feeling, is jumping up and down is put those who did not do will hurry well is just the whole person will become very nervous mood not feel nervous the whole person will become the whole action, the above will become like is that soon, but the heart, is actually. (M5)  Quote 26: For a while, I had problems with the perineum (self-doubt and unconfidence). There are always very afraid to the stage are very afraid of such a thing again, when the time comes on stage (birth) situation.(Affect the mood at work). (M11)  Quote 27: If the kind of tension at work may affect more than 1,2 months, that half a year all have to be careful. (M12) | 20 |
| **Theme 3: Exploring effective coping strategies** | |  |
| **Subtheme 1: Need time to recover** | Quote 28: It may take one to two months, and it scares me to think about it from time to time *“forced smile”.* If you face this every day, you need a adjustment. Then keep a self-comfort, keep self-regulation. (M1)  Quote 29: It may take a few days to forget something like this, and then you will remember it from time to time, but the mood swings will not be so great. (M6)  Quote 30:About a week or two, there may also be such negative mood at work. Then the heart (afraid of this kind of thing) feel, (afraid of this happening again) will be more nervous, affect their next things do not do well. The pressure of the incident in a week or two has affected the quality of my work. However, if it is unfair to my colleagues that the quality of work due of this matter is bad, so I will try to adjust myself, be more cautious in my work and do things well. (M8) | 15 |
| **Subtheme 2: Released stress** |  |  |
| Distract attention | Quote 31: Is to collect more knowledge, is more knowledge about this aspect to enrich themselves. In the future, clinically, I think I will work harder and try my best to reduce or eliminate the occurrence of such events and do my own part. On the other hand, will I spend my spare time after work as some activities I like? Can I discuss with my colleagues and conclude what things that need to be improved. Strengthen their own (ability), let oneself not so afraid.(M7)  Quote 32: I just bake, eat or go shopping, and when the pressure is too much, we will travel to distract ourselves. (M10)  Quote 33: Exercise, walking, walking and running are like this. In addition to self-regulation, sometimes I will write a diary, but less. (M13) | 37 |
| Ask for accompany | Quote 34: But to sum up the experience, well, and if I want to talk more with my colleagues, I think it's good, maybe that's it, I don't have more experience, uh. (M2)  Quote 35: Just self-regulation, sometimes I will write a diary. I am wondering, do you have some ways to help me? (M13)  Quote 36: And then I think if that traumatic birth happens, we don't care enough us midwives, and we don't provide psychological support. When something happened, it was more about accountability, rarely asking midwives about their psychological feelings. In fact, sometimes we are also very confused, but also want someone to comfort us. (M13) | 8 |
